# Common *TGFβ2*, *BMP4*, and *FOXC1* variants are not associated with primary open-angle glaucoma

**Published:** 2012-06-13

**Authors:** Soo Park, Yalda Jamshidi, Daniela Vaideanu, Scott Fraser, Jane C. Sowden

**Affiliations:** 1 Tennent Institute of Ophthalmology, Gartnavel General Hospital, Glasgow, UK; 2 Human Genetics Research Centre, St George's University of London, UK; 3 Ophthalmology Department, Sunderland Eye Infirmary, Sunderland, UK; 4 Developmental Biology Unit, University College London Institute of Child Health and Great Ormond Street Hospital for Children NHS Trust, London, UK

## Abstract

**Purpose:**

Primary open-angle glaucoma (POAG) is a common but complex disease with a strong genetic component. Notably, few genes have been robustly associated with POAG. An obvious group of genes to test as susceptibility factors for POAG are the developmental genes forkhead box C1 (*FOXC1*), transforming growth factor-beta 2 (*TGFβ2*), and bone morphogenic protein 4 (*BMP4*). These genes are known to play important roles in the normal morphogenesis of the anterior segment and/or have been implicated in intra-ocular pressure (IOP) regulation and trabecular meshwork function. This study investigates the role of *FOXC1*, *TGFβ2*, and *BMP4* in POAG.

**Methods:**

The contribution of common genetic variation at the *FOXC1*, *TGFβ2*, and *BMP4* loci to risk of POAG was investigated in a case-control association study in 330 British Caucasian individuals comprised of 272 high-tension glaucoma (HTG) and 58 ocular hypertension (OHT), and 276 matched controls.

**Results:**

All the single nucleotide polymorphisms (SNPs) were in Hardy–Weinberg equilibrium and genotyping success rate was >92% for all SNPs. With the exception of a weak association between the *BMP4* tagging SNP rs2761884 and the combined patient group HTG+OHT that did not withstand permutation testing (uncorrected p=0.0400, corrected p=0.1320), no associations (p<0.05) were identified between the patient groups (HTG and OHT) and *FOXC1*, *TGFβ2*, and *BMP4* alleles and haplotypes compared to the control group.

**Conclusions:**

This is the first association analysis of *FOXC1*, *TGFβ2*, and *BMP4* and POAG. These genes were selected as candidate genes for POAG because of their biologic roles. No significant associations were identified between *FOXC1*, *TGFβ2*, and *BMP4* alleles and haplotypes and POAG. The lack of association suggests that common variation in these genes do not have a significant role in the pathogenesis of POAG among British Caucasian subjects.

## Introduction

Glaucoma is a complex heterogeneous disorder characterized by an optic neuropathy in which progressive degeneration of retinal ganglion cells leads to excavation of the optic nerve head and to visual field loss. It is a major cause of visual impairment and blindness worldwide affecting approximately 67 million people [1]. Primary open-angle glaucoma (POAG), the most common form of glaucoma, has an estimated prevalence of 1.2% for the age group 40 to 89 years in the white UK population [2]. POAG is predominately composed of high-tension glaucoma, where the intraocular pressure (IOP) is raised (IOP >21 mmHg). Normal-tension glaucoma (NTG), which is another important but less common subgroup of POAG, is an optic neuropathy similar to HTG but the IOP levels are within the statistically normal range (IOP ≤21 mmHg). On the other hand, individuals with ocular hypertension (OHT) have raised IOP without clinical signs of glaucomatous optic neuropathy; however, OHT is an important risk factor for POAG, and 10% will convert to POAG over a 10-year period [3].

Although the proportion of genetically attributable cases of POAG is unknown, there is increasing evidence to suggest POAG has a significant heritable basis. Population-based studies have shown that a positive family history is an important risk factor for POAG. The relative risk of developing POAG among individuals with a positive family history varies from 3 to 10-fold [4,5]. Further evidence that genetic factors are important is supported by twin studies which have shown a higher degree of concordance among monozygotic twin [6,7]. In particular, Gottfredsdottir et al. [6] showed the concordance of open angle glaucoma in monozygotic twin pairs was significantly higher at 98% compared to their spouses (70%). Moreover, the prevalence of POAG is highest in black populations and lowest in northern Asian populations; these ethnic differences may be attributable to genetics among other factors [8]. Genetic linkage studies among rare pedigrees with Mendelian patterns of adult-onset POAG inheritance have identified 14 genetic loci (GLC1A-N) [9-19]. However, only 3 genes (myocilin, trabecular meshwork inducible glucocorticoid response [*MYOC*], optineurin [*OPTN*], and WD repeat domain 36 [*WDR36*]) have shown to be robustly associated with POAG in the general population. Furthermore, only *MYOC* (GLC1A) is established as directly causative, mutations of which account for 5% of POAG, while due to conflicting results the exact roles of *OPTN* (GLC1E) and *WDR36* (GLC1G) in POAG remain uncertain [20,21]. A recent genome-wide association study involving 590 affected individuals with advanced POAG and 3,956 controls, has identified susceptible loci at transmembrane and coiled-coil domain-containing protein 1 (*TMCO1*) and CDKN2B antisense RNA 1 (non-protein coding; *CDKN2B-AS1*) for POAG [22]. Based on current knowledge, it is probable that POAG is a genetically heterogeneous disorder caused by the interaction between several genetic and environmental factors.

Genes that cause developmental glaucoma [23], with the exception of the cytochrome P450, family 1, subfamily B, polypeptide 1 (*CYP1B1*) and LIM homeobox transcription factor 1, beta (*LMX1B*) genes, have yet to be assessed as genetic susceptibility factors for POAG. *CYP1B1* causes primary congenital glaucoma and is also involved in cases of juvenile open-angle glaucoma [24]. A recent study has implicated a *CYP1B1* polymorphism as a susceptibility factor for POAG [25]. *LMX1B* mutations, on the other hand, cause dominantly-inherited Nail-Patella Syndrome (NPS; OMIM 161200) in which approximately 33% of patients develop glaucoma [26]. More importantly, *LMX1B* haplotypes have shown to influence susceptibility to POAG [27].

Developmental glaucoma refers to glaucomas that are associated with developmental malformations of the anterior segment of the eye [28]. Anterior segment dysgenesis (ASD) may lead to incomplete development, or dysfunction, of the structures that form the aqueous drainage pathway, and can result in IOP elevation secondary to aqueous outflow obstruction, predisposing to glaucoma [23]. Existing studies indicate that developmental glaucoma genes forkhead box C1 (*FOXC1*), transforming growth factor-beta 2 (*TGFβ2*), and bone morphogenic protein 4 (*BMP4*) are strong candidate genes for POAG susceptibility. *Foxc1* is expressed in the developing trabecular meshwork (TM) [29] whereas *TGFβ2* and *BMP4* are expressed in the adult human TM [30,31]. All of these genes cause developmental malformation of the anterior segment [29,32,33]. The essential role of these developmental glaucoma genes for the development of the anterior segment and in the development of TM implies that *FOXC1*, *TGFβ2*, and *BMP4* are crucial for the normal development of drainage structures and preservation of normal IOP. This idea is supported by targeted heterozygous mutation in animal models resulting in malformation of the drainage structures [29,32,33] with a high incidence of glaucoma ranging from 40%–75% or above [33,34].

Elevated levels of TGFβ2 have been found in POAG patients. In addition, studies have shown that raised IOP in POAG is as a result of increased resistance to aqueous outflow [35] and this is associated with biochemical and morphological changes in the TM [36]. There is an accumulation of extracellular matrix (ECM) in the TM of glaucoma patients, and this may be as a result of disruption of the balance between ECM deposition and degradation [36]. In vitro studies have shown that *TGFβ2* and *BMP4* act in concert to maintain a balance between ECM deposition and degradation, and may play an important role in glaucoma pathogenesis through mis-regulation of ECM synthesis and cross-linkage of ECM components of the TM [30].

Since *Foxc1* is expressed in the developing embryonic TM [29], mutations or altered expression of *FOXC1* could interfere with normal function of the tissue and lead to increased risk of glaucoma. Although expression of *FOXC1* is yet to be studied in adults, it is highly possible that continued expression of the abnormal gene product (from age-related, subclinical mutations) throughout life, or altered levels of expression of *FOXC1* could interfere with normal function of the TM, thereby leading to increased risk of glaucoma through the effects of raised IOP. This notion is supported by the fact that glaucoma associated with mutations in the developmental glaucoma genes can present at any time from birth to adulthood, and in some instances above 70 years of age [37]. Furthermore, in some affected family members with glaucoma as a result of *FOXC1* mutations, the anterior segment malformation may be very subtle, and easily missed in clinical examination [37,38], a feature more in keeping with POAG. In addition, the risk of developing glaucoma is not related to the severity of the phenotype [37], suggesting that subtle dysfunction of the angle drainage structures may be contributing toward glaucoma [39].

It is thus plausible that these developmental glaucoma genes contribute to age-related open angle glaucoma, where the ocular drainage structures have abnormalities that are not clinically visible but which cause dysfunction with age. We hypothesize that sub-clinical mutations/polymorphisms in *FOXC1*, *TGFβ2*, and *BMP4* may produce subtle and undetected abnormalities in anterior segment structure and function, which predispose to glaucomatous optic neuropathy through the effects of raised IOP and may be a significant susceptibility factor for the development of OHT and POAG.

In this study, we assess whether variant alleles of *FOXC1*, *TGFβ2*, and *BMP4* play a role in the general population. A case-control genetic association study was performed to compare the prevalence of *FOXC1*, *TGFβ2*, and *BMP4* tagging single nucleotide polymorphisms (tSNPs) in three groups, HTG, OHT, and a normal control group. Haplotypes in *FOXC1*, *TGFβ2*, and *BMP4* were identified and their prevalence assessed in patients with glaucomatous optic neuropathy (HTG patients) and in patients with raised IOP (HTG and OHT patients).

## Methods

### Recruitment of patients

All of the participating subjects were recruited from glaucoma outpatient clinics at the Sunderland Eye Infirmary in the North-East of England, UK, a secondary ophthalmology referral center. The research followed the tenets of the Declaration of Helsinki. Informed consent was obtained from all participants after the nature and possible consequences of the study were explained. The study had Local Research Ethics Committee approval. A cohort of HTG cases (n=272), and unrelated controls (n=276) matched for ethnicity, age and sex were recruited to the study. Cases with OHT (n=58) were also collected. All cases (n=330) and controls were of British Caucasian descent.

Control participants, either accompanying spouses or friends of individuals with glaucoma, were recruited randomly. All controls underwent a complete ophthalmic examination to exclude individuals with glaucoma from the control group, and were confirmed to have no visual complaints and IOP of <22 mmHg with a normal disc appearance. Individuals with a family history of glaucoma were excluded.

All case subjects underwent a complete ophthalmic examination as previously described (Park et al. [27]) including best visual acuity, and visual field testing using a Humphrey SITA standard 24–2 perimetry (Carl Zeiss Meditec AG, Jena, Germany), slit lamp examination of the anterior segment (including gonioscopy), measurement of IOP by Goldmann applanation tonometer, posterior segment examination of the retina and optic disc following pupil dilation and measurement of the cup-disc ratio (CDR). The clinical diagnosis (including assessment of visual fields) was made by the same consultant with a special interest in glaucoma and experience in anterior segment phenotyping. This ensured exclusion of individuals with glaucoma from the control group and made certain that cases were correctly classified either as HTG and OHT. Central corneal thickness (CCT) data was not collected for the cases and controls in the current study since a pachymeter was not available at the time when this study was performed. Adult individuals with a diagnosis of HTG or OHT after the age of 40 years were enrolled based on the following clinical criteria:

Presence of glaucomatous optic neuropathy (defined by loss of neuroretinal rim) with compatible and reproducible visual field loss for HTG, and absence of detectable glaucomatous damage or field loss for OHT. All of the visual field tests showed reproducible field defects that were compatible with the degree of glaucomatous cupping of the optic nerve head (defined by loss of neuroretinal rim), and were ensured to have a satisfactory reliability score of ≤20% fixation loss, false positive of ≤33% and/or false negative of ≤33%;

Open drainage angles on gonioscopy;

IOP consistently ≥22 mmHg on diurnal testing for HTG and OHT. To be certain that the participants were correctly assigned to the appropriate case groups, individuals with borderline IOPs (21–23 mmHg) were excluded from this study;

Absence of a secondary cause for glaucomatous optic neuropathy;

Absence of non-glaucomatous field losses and disc changes (i.e., high myopia).

### Selection and analysis of single nucleotide polymorphisms

We employed a tagging single nucleotide polymorphism approach to screen *FOXC1*, *TGFβ2*, and *BMP4* genes including 10 kb of upstream and downstream flanking region in patient and control groups using tSNPs selected from the HapMap database (HapMap Data Release #22/Phase II Apr 2007; Centre d'Etude du Polymorphisme Humain [CEPH] population). Genotypes of 90 CEU The Sequenom iPLEX^TM^ Assay MassARRAY® (Sequenom, San Diego, CA) was used for high-throughput SNP genotyping (details of primer information used for SNP genotyping provided in Appendix 1). Allele frequencies for each SNP were tested for agreement with Hardy–Weinberg expectations (p>0.05) using a χ^2^ goodness-of-fit test.

**Figure 1:**
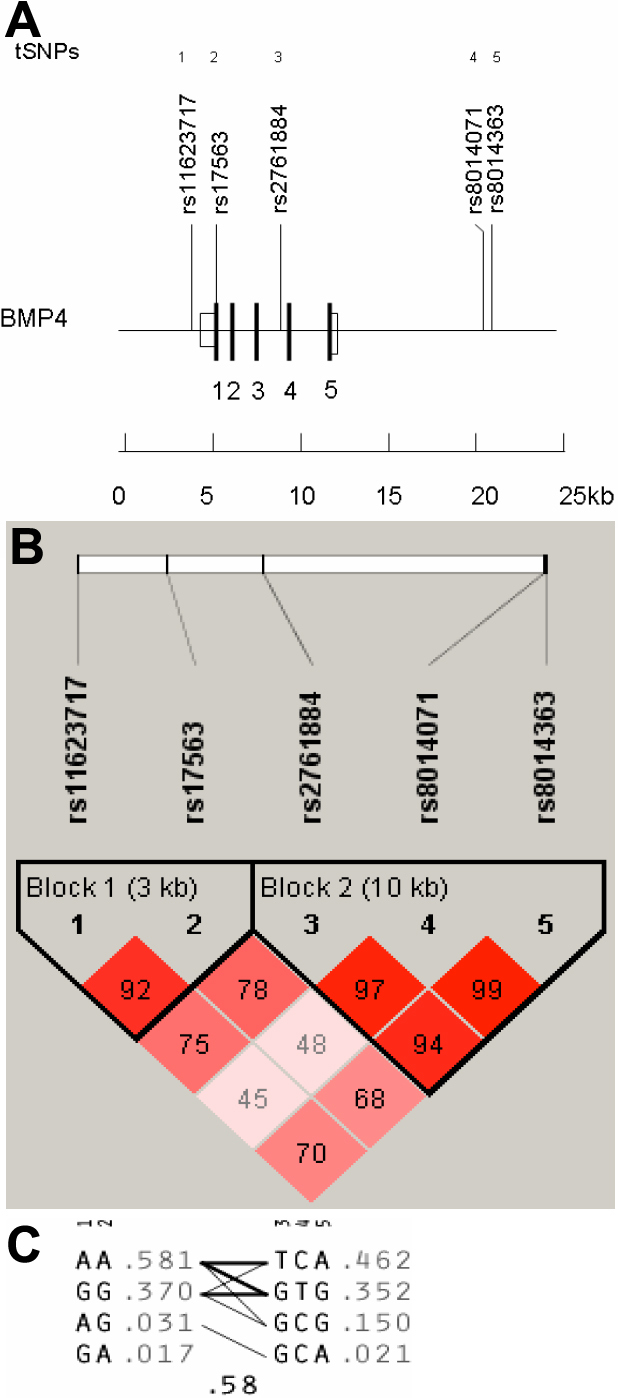
Linkage disequilibrium plot of *BMP4* region HapMap SNPs. **A**: The relative position of the 5 tSNPs in *BMP4* spanning a region of 17,443 bp (Chr14: 53483882–53501325). Four coding exons are indicated as solid boxes and numbered accordingly. Untranslated exons are shown as open boxes. **B**: Diagram of block structure of *BMP4* generated using Haploview v.4.0. LD plots were identified by strong LD. Depth of red/pink color indicates the computed pairwise *D*' value; deeper pink indicates a higher *D*' value. **C**: The selected tSNPs and estimated haplotype frequencies in the two major haplotype blocks are shown. Marker numbers and arrows above the haplotypes indicate tSNPs. The frequency of each haplotype within a block is given to the right of the haplotype. The thickness of the lines connecting the haplotypes across blocks represents the relative frequency (i.e., high [*thick*] versus low [*thin*]) with which a given haplotype is associated with the haplotype in the neighboring block.

**Figure 2:**
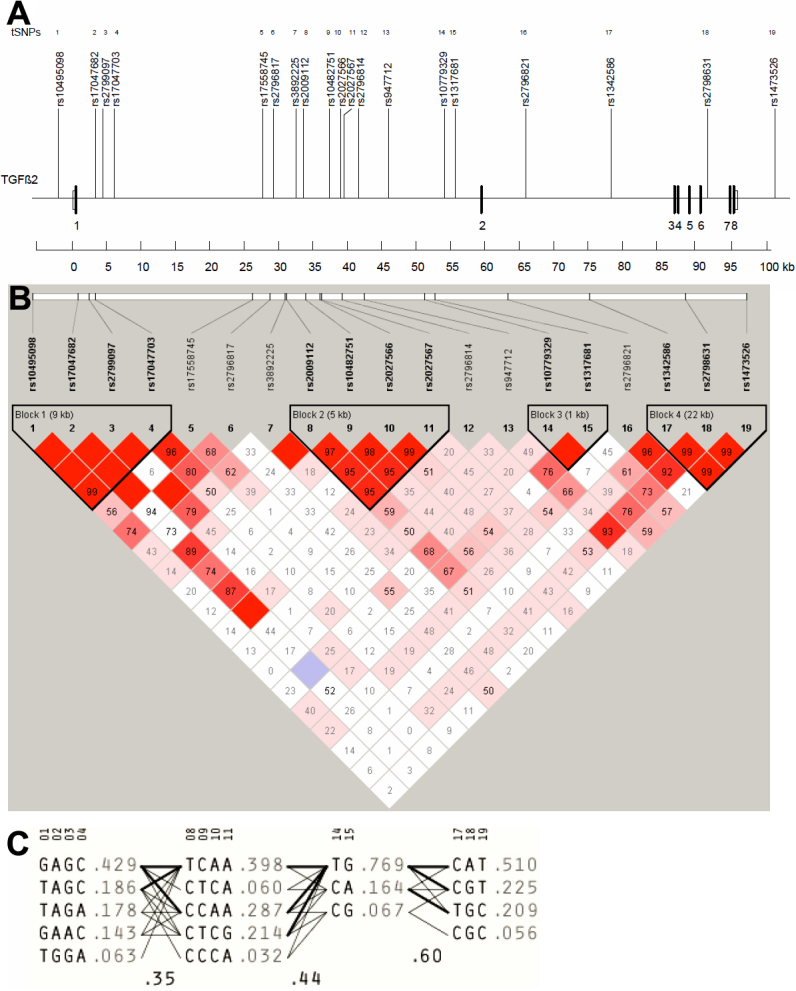
Linkage disequilibrium plot of *TGFβ2* region HapMap SNPs. **A**: The relative position of the remaining 19 tSNPs in *TGFβ2* (labeled above with the respective haplotype) spanning a region of 104,437 bp (Chr1: 216582933–216687370). Eight coding exons are indicated as solid boxes and numbered accordingly. Untranslated exons are shown as open boxes. **B**: Diagram of block structure of *TGFβ2* generated using Haploview v.4.0.LD plots were identified by strong LD. Depth of red/pink color indicates the computed pairwise *D*' value; deeper pink indicates a higher *D*' value. **C**: The selected tSNPs and estimated haplotype frequencies in the four major haplotype blocks (1–4) are shown. Marker numbers above the haplotypes indicate tSNPs. The frequency of each haplotype within a block is given to the right of the haplotype. The thickness of the lines connecting the haplotypes across blocks represents the relative frequency (i.e., high [*thick*] versus low [*thin*]) with which a given haplotype is associated with the haplotype in the neighboring block.

**Figure 3:**
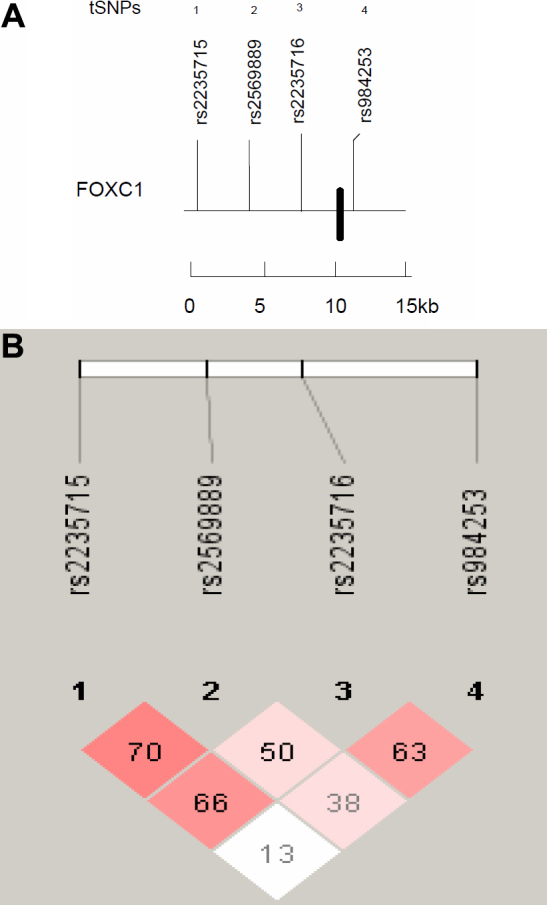
Linkage disequilibrium plot of *FOXC1* region HapMap SNPs. **A**: The relative position of the remaining 4 tSNPs (labeled above with the respective haplotype) spanning a region of 14,587 bp in *FOXC1* (Chr6:1543941–1558528). The coding exon is indicated as a solid box. **B**: Diagram of block structure of *FOXC1* generated using Haploview v.4.0 showing absence of common haplotype due to low LD between tSNPs. Depth of red/pink color indicates the computed pairwise *D*' value; deeper pink indicates a higher *D*' value.

A total of 5 tSNPs for *BMP4* spanning a region of 15,272 bp (including the 4,814 bp *BMP4* gene), 19 tSNPs for *TGFβ2,* spanning a region of 98,075 bp (including the 95,108 bp *TGFβ2* gene), and 4 tSNPs for *FOXC1* spanning a region of 12,012 bp (including the 1,661 bp *FOXC1* gene) were selected from the HapMap database and genotyped in all individuals (Figure 1, Figure 2, and Figure 3).

### LD and haplotype structure of the *FOXC1*, *TGFβ2*, and *BMP4* genomic region

Haplotypes were inferred using Haploview v.4.0, and associations between tSNP or haplotype and glaucoma were investigated. The method of Gabriel et al. [40], as implemented in Haploview, was used to construct LD blocks from tSNPs with minor allele frequencies (MAF) ≥5%. LD between tSNPs was measured by the pairwise *D*' statistic and the LD structure was examined using the 80% confidence bounds of *D*' to define sites of historical recombination between tSNPs.

Haplotypes were constructed from genotype data in the full-size case-control panel within blocks by using an accelerated expectation-maximization algorithm method [41]. In each haplotype block, common haplotypes with frequencies ≥1% were inferred that accounted for >98% of the chromosomes. Differences in genotype and haplotype frequencies between cases and controls were determined using a χ^2^ distribution with 2 degrees of freedom. Permutation testing was performed to calculate corrected p-values for multiple testing with 1,000 simulations. Odds ratios (ORs) were calculated using THESIAS v.3.1 with 95% confidence intervals (CIs) for each genotype with the respective wild type as the reference. Thesias is based on the maximum likelihood model described in Tregouet et al. [42].

Using the Stata built-in power and sample size functions (Stata Statistical Software: Release 8.0; Stata Corporation, College Station, TX), a power of 80% was estimated using the sample size of 276 controls and 330 cases, to identify a difference in genotype and allele frequency between 10%–18% at a significance level of p<0.05 between the controls and cases.

## Results

Among the cases, 272 (74.3%) were classified as HTG, and 58 (15.6%) as OHT (Table 1). All tSNPs (Figure 1, Figure 2, and Figure 3) conformed to Hardy–Weinberg equilibrium and the genotyping success rate varied from 92.0% and 99.0%. The pairwise LD matrix revealed 2 main haplotype blocks for *BMP4* (Figure 1) and 4 haplotype blocks for *TGFβ2* (Figure 2). Haplotype analysis of *FOXC1* revealed no common haplotype between patients and variants of *FOXC1* (Figure 3) due to lack of LD between SNPs.

**Table 1:** Comparison of age, sex, mean CDR and mean IOP between HTG, OHT, HTG+OHT group (with raised IOP) and the control group.

**Characteristic**	**OHT**	**HTG**	**Controls**	**Cases combined (OHT+HTG)**	**p^1^**
N	58	272	276	330	
Age (SD)	*65.19 (11.494)	71.17 (10.448)	70.76 (9.313)	70.12 (10.863)	0.437
Sex % Male	41.4	*54.4	42.8	52.1	0.022
Mean CDR (SD)	0.3629 (0.138)	*0.7175 (0.182)	0.2120 (0.231)	0.6552 (0.221)	<0.0001
Mean IOP (SD)	*27.16 (4.021)	*29.13 (5.506)	15.45 (2.352)	28.79 (5.326)	<0.0001

* indicates significant difference (p<0.05) between controls and the separate case groups (HTG, OHT). p^1^ indicates significant difference between controls and the HTG+OHT group. p values were calculated using independent samples *t*-test, except a χ^2^ test was used for sex, using SPSS, version 15 (SPSS Inc., Chicago, IL).

### Lack of association between SNPs in *TGFβ2, BMP4*, and *FOXC1* and POAG

The allele frequencies of the 19 tSNPs in *TGFβ2*, 5 tSNPs in *BMP4* and 4 tSNPs in *FOXC1* between the 3 separate case groups (HTG, OHT, HTG+OHT) and the controls were assessed. No significant associations were found between *TGFβ2*, *BMP4*, and *FOXC1* and glaucoma (Table 2 [A], Table 3 [A], Table 4), except between the combined group HTG+OHT and *BMP4* (Table 3 [A]) where there was a weak association which did not withstand permutation testing (uncorrected p=0.040, corrected p=0.1320, OR 1.26). In addition, these tSNPs were analyzed under 3 different genetic models (dominant, co-dominant and recessive models) and no significant associations were identified (data not shown).

**Table 2:** No significant associations identified between *TGFβ2* SNPs and haplotypes and POAG.

**A**						
**SNP ID**	**Allele**	**Phenotype**	**Case counts (%)**	**Control counts (%)**	**χ^2^**	**Uncorrected p-value**
rs10495098	G	HTG	297 (56.2)	311 (56.8)	0.03	0.8619
	G	OHT	72 (65.5)	311 (56.8)	2.85	0.0912
	G	HTG+OHT	370 (57.8)	311 (56.8)	0.14	0.7125
rs17047682	G	HTG	39 (7.4)	31 (5.7)	1.32	0.2502
	G	OHT	5 (4.5)	31 (5.7)	0.22	0.6399
	G	HTG+OHT	44 (6.9)	31 (5.7)	0.77	0.3819
rs2799097	A	HTG	69 (13.3)	80 (15.0)	0.67	0.4108
	A	OHT	17 (16.0)	80 (15.0)	0.07	0.7934
	A	HTG+OHT	86 (13.7)	80 (15.0)	4.21	0.0601
rs17047703	A	HTG	140 (26.8)	127 (23.2)	1.89	0.1685
	A	OHT	21 (19.1)	127 (23.2)	0.72	0.3956
	A	HTG+OHT	161 (25.5)	127 (23.2)	0.84	0.3591
rs17558745	T	HTG	191 (36.6)	169 (31.2)	3.47	0.0623
	T	OHT	30 (28.0)	169 (31.2)	0.01	0.9134
	T	HTG+OHT	221 (35.1)	169 (31.2)	1.99	0.1579
rs2796817	G	HTG	70 (13.2)	83 (15.1)	0.78	0.3749
	G	OHT	19 (17.3)	83 (15.1)	0.33	0.5633
	G	HTG+OHT	89 (13.9)	83 (15.1)	0.34	0.5623
rs3892225	G	HTG	119 (22.5)	106 (19.3)	1.74	0.1873
	G	OHT	20 (18.2)	106 (19.3)	0.07	0.7904
	G	HTG+OHT	139 (21.8)	106 (19.3)	1.14	0.2855
rs2009112	C	HTG	215 (40.6)	222 (40.4)	0.01	0.946
	C	OHT	42 (38.2)	222 (40.4)	0.18	0.6698
	C	HTG+OHT	257 (40.2)	222 (40.4)	0.01	0.9420
rs10482751	C	HTG	145 (27.6)	153 (27.8)	0.01	0.9265
	C	OHT	30 (27.3)	153 (27.8)	0.01	0.9071
	C	HTG+OHT	175 (27.5)	153 (27.8)	0.01	0.9076
rs2027566	C	HTG	167 (31.5)	172 (31.4)	0.00	0.9654
	C	OHT	35 (31.8)	172 (31.4)	0.01	0.9292
	C	HTG+OHT	202 (31.6)	172 (31.4)	0.00	0.9482
rs2027567	G	HTG	116 (22.1)	124 (22.5)	0.04	0.8463
	G	OHT	22 (20.0)	124 (22.5)	0.35	0.5571
	G	HTG+OHT	138 (21.7)	124 (22.5)	0.12	0.7258
rs2796814	G	HTG	132 (25.1)	118 (21.6)	1.82	0.1776
	G	OHT	26 (23.6)	118 (21.6)	0.22	0.6398
	G	HTG+OHT	158 (24.8)	118 (21.6)	1.71	0.1905
rs947712	T	HTG	185 (35.6)	191 (35.1)	0.03	0.8735
	T	OHT	41 (37.3)	191 (35.1)	0.19	0.6655
	T	HTG+OHT	226 (35.9)	191 (35.1)	0.07	0.7854
rs10779329	C	HTG	124 (23.6)	119 (21.7)	0.53	0.4667
	C	OHT	31 (28.2)	119 (21.7)	2.18	0.1401
	C	HTG+OHT	155 (24.4)	119 (21.7)	1.17	0.2800
rs1317681	A	HTG	84 (15.9)	89 (16.4)	0.04	0.8409
	A	OHT	20 (18.5)	89 (16.4)	0.30	0.5830
	A	HTG+OHT	104 (16.4)	89 (16.4)	0.00	0.9970
rs2796821	T	HTG	147 (27.8)	144 (26.2)	0.38	0.5396
	T	OHT	33 (30.0)	144 (26,2)	0.68	0.4093
	T	HTG+OHT	180 (28.2)	144 (26.2)	0.61	0.4331
rs1342586	C	HTG	106 (20.0)	125 (22.8)	1.26	0.2609
	C	OHT	19 (17.3)	125 (22.8)	1.64	0.1999
	C	HTG+OHT	125 (19.5)	125 (22.8)	1.91	0.1669
rs2798631	G	HTG	255 (48.7)	267 (48.9)	0.01	0.9382
	G	OHT	55 (50.0)	267 (48.9)	0.04	0.8334
	G	HTG+OHT	310 (48.9)	267 (48.9)	0.00	0.9986
rs1473526	C	HTG	138 (26.1)	150 (27.3)	0.18	0.6734
	C	OHT	26 (23.6)	150 (27.3)	0.62	0.4311
	C	HTG+OHT	64 (25.7)	150 (27.3)	0.37	0.5413
**B**						
**Block**	**Haplotype**	**All subjects (%)**	**Case (%)**	**Control (%)**	**χ^2^**	**p-value**
**1**						
(tSNPs 1 to 4)	GAGC	42.2	42.9	41.6	0.19	0.6657
	TAGC	18.8	17.3	20.2	1.56	0.2112
	TAGA	18.3	19.1	17.5	0.49	0.4841
	GAAC	14.2	13.4	15.1	0.67	0.4123
	TGGA	6.5	7.4	5.6	1.39	0.2379
**2**						
(tSNPs 8 to 11)	TCAA	40.1	40.1	40.1	0.00	0.9891
	CCAA	28.4	28.3	28.5	0.01	0.9386
	CTCG	21.5	21.2	21.9	0.08	0.7746
	CTCA	6.0	6.3	5.7	0.20	0.6592
	CCCA	3.0	2.9	3.2	0.10	0.7473
**3**						
(tSNPs 14 to 15)	TG	77.4	76.5	78.3	0.47	0.4950
	CA	16.2	16.0	16.5	0.05	0.8175
	CG	6.3	7.5	5.2	2.32	0.1280
**4**						
(tSNPs 17 to 19)	CAT	51.1	51.1	51.1	0.00	0.9927
	CGT	22.1	22.7	21.5	0.21	0.6462
	TGC	21.2	19.8	22.6	1.28	0.2581
	CGC	5.5	6.2	4.7	1.16	0.2809
**C**						
**Block**	**Haplotype**	**All subjects (%)**	**Case (%)**	**Control (%)**	**χ^2^**	**p-Value**
**1**						
(tSNPs 1 to 4)	GAGC	42.9	44.0	41.6	0.70	0.4035
	TAGC	18.6	17.1	20.3	1.92	0.1664
	TAGA	17.8	18.1	17.4	0.10	0.7500
	GAAC	14.3	13.7	15.1	0.48	0.4866
	TGGA	6.3	6.9	5.6	0.82	0.3659
**2**						
(tSNPs 8 to 11)	TCAA	39.8	39.6	40.1	0.03	0.8680
	CCAA	28.7	28.8	28.5	0.01	0.9220
	CTCG	21.4	20.9	21.9	0.15	0.6992
	CTCA	6.0	6.3	5.7	0.22	0.6401
	CCCA	3.2	3.1	3.2	0.00	0.9491
**3**						
(tSNPs 14 to 15)	TG	76.9	75.7	78.3	1.08	0.2979
	CA	16.4	16.4	16.5	0.00	0.9485
	CG	6.7	7.9	5.2	3.44	0.0636
**4**						
(tSNPs 17 to 19)	CAT	51.0	50.9	51.1	0.01	0.9415
	CGT	22.5	23.3	21.5	0.54	0.4606
	TGC	20.9	19.4	22.6	1.89	0.1691
	CGC	5.6	6.3	4.7	1.29	0.2556

A: Distribution of *TGFβ2* tSNPs between HTG, OHT and HTG+OHT, compared to the wild type control group. B: Distribution of *TGFβ2* haplotypes showing no significant associations between HTG cases and controls. C: Distribution of *TGFβ2* haplotypes showing no significant associations between HTG+OHT cases and controls.

**Table 3:** No significant associations identified between *BMP4* SNPs and haplotypes and POAG except between the combined group HTG+OHT and *BMP4* where there was a weak association.

**A**						
**SNP ID**	**Allele**	**Phenotype**	**Case counts (%)**	**Control counts (%)**	**χ^2^**	**Uncorrected p- value**
rs11623717	A	HTG	197 (62.4)	219 (60.0)	0.63	0.4264
	A	OHT	68 (61.8)	329 (60.0)	0.12	0,7274
	A	HTG+OHT	395 (62.3)	329 (60.0)	0.64	0.4251
rs17563	A	HTG	315 (61.5)	298 (58.0)	1.34	0.2467
	A	OHT	60 (60.0)	298 (58.0)	0.14	0.7073
	A	HTG+OHT	375 (61.3)	298 (58.0)	1.26	0.2610
rs2761884	T	HTG	265 (50.2)	243 (44.3)	3.69	0.0548
	T	OHT	56 (50.9)	243 (44.3)	1.59	0.2069
	**T**	**HTG+OHT**	**321 (50.3)**	**243 (44.3)**	**4.21**	**0.0401**
rs8014071	C	HTG	342 (64.8)	344 (63.0)	0.36	0.5463
	C	OHT	74 (67.3)	344 (63.0)	0.72	0.3956
	C	HTG+OHT	416 (65.2)	344 (63.0)	0.62	0.4312
rs8014363	A	HTG	248 (49.6)	235 (47.6)	0.41	0.5222
	A	OHT	52 (48.1)	235 (47.6)	0.01	0.9134
	A	HTG+OHT	300 (49.3)	235 (47.6)	0.34	0.5585
**B**						
**Block**	**Haplotype**	**All subjects (%)**	**Case Counts (%)**	**Control Counts (%)**	**χ^2^**	**p-Value**
**1**						
(tSNP 1 to 2)	AA	57.9	59.1	56.9	0.53	0.4652
	GG	37.1	35.3	38.9	1.47	0.2255
	AG	3.2	3.2	3.2	0.00	0.9555
	GA	1.7	2.4	1.1	2.73	0.0985
**2**						
(tSNP 3 to 5)	TCA	46.1	48.0	44.3	1.55	0.2131
	GTG	35.6	34.2	36.9	0.87	0.3506
	GCG	14.9	13.8	5.9	0.92	0.3389
	GCA	2.2	1.6	2.8	1.86	0.1722
**C**						
**Block**	**Haplotype**	**All subjects (%)**	**Case counts (%)**	**Control counts (%)**	**χ^2^**	**p-Value**
**1**						
(tSNP 1 to 2)	AA	58.1	59.2	56.9	0.63	0.4270
	GG	37.0	35.5	38.9	1.47	0.2252
	AG	3.1	3.1	3.2	0.01	0.9151
	GA	1.7	2.3	1.1	2.62	0.1055
**2**						
(tSNP 3 to 5)	TCA	46.2	47.9	44.2	1.58	0.2084
	GTG	35.2	33.8	36.9	1.29	0.2565
	GCG	15.0	14.3	15.9	0.62	0.4322

A: Distribution of *BMP4* tSNPs between HTG, OHT and HTG+OHT, compared to the wild type control group. tSNPs that are significantly distributed (p<0.05) are high lighted in bold. B: Distribution of *BMP4* haplotypes showing no significant associations between HTG cases and controls. C: Distribution of *BMP4* haplotypes showing no significant associations between HTG+OHT cases and controls.

**Table 4:** Distribution of *FOXC1* tSNPs between HTG, OHT and HTG+OHT, compared to the wild type control group.

**SNP ID**	**Allele**	**Phenotype**	**Case counts (%)**	**Control counts (%)**	**χ^2^**	**Uncorrected p-value**
rs2235715	T	HTG	192 (88.9)	320 (83.3)	3.41	0.0648
	T	OHT	52 (78.8)	320 (83.3)	0.81	0.3675
	T	HTG+OHT	244 (86.5)	320 (83.3)	1.28	0.2585
rs2569889	C	HTG	260 (49.6)	259 (48.7)	0.09	0.7614
	C	OHT	53 (47.3)	259 (48.7)	0.07	0.7931
	C	HTG+OHT	313 (49.2)	259 (48.7)	0.03	0.8569
rs2235716	T	HTG	363 (69.3)	350 (65.1)	2.14	0.1433
	T	OHT	69 (62.7)	350 (65.1)	0.22	0.6416
	T	HTG+OHT	432 (68.1)	350 (65.1)	1.25	0.2643
rs984253	T	HTG	185 (35.7)	183 (34.4)	0.20	0.6550
	T	OHT	39 (34.8)	183 (34.4)	0.01	0.9318
	T	HTG+OHT	224 (35.6)	183 (34.4)	0.17	0.6804

### Absence of association between haplotypes in *TGFβ2* and *BMP4* and HTG

The difference in the distribution of all common haplotypes in *TGFβ2* and *BMP4* (see Table 2 [B] and Table 3 [B]) between individuals with HTG and controls was assessed (but not for *FOXC1* as common haplotypes were not present) and no significant haplotype associations were identified for each haplotype blocks. The absence of common haplotypes between patients and *FOXC1* can be explained by the small size of the FOXC1 gene (3,447 bp), which makes it less likely that haplotype blocks are present in such a small region.

### Absence of association between haplotypes in *TGFβ2* and *BMP4,* and raised IOP (HTG+OHT)

Here, assessment for possible common haplotype associations between combined raised IOP patient group (HTG+OHT) and the controls were performed for *TGFβ2* and *BMP4* (see Table 2 [C] and Table 3 [C]) but not for *FOXC1* as common haplotypes were not present and no significant haplotype effects for each haplotype block were found.

## Discussion

Despite recent progress in identifying genes associated with glaucoma, the contribution of genetics to the pathogenesis of POAG continues to remain unclear. Given the relatively high prevalence of POAG within the normal population, and the fact that it is amendable to treatment when detected early, identification of genetic risk factors would offer the prospect of early POAG diagnosis, in addition to the tailoring of appropriate treatments to those who would be most likely to benefit. However, such screening programs are currently limited by the paucity of the identified causative genes [43] and identification of the most significant disease-associated alleles in different populations is of paramount importance.

Recent work has started to investigate whether analysis of genetic risk in glaucoma can be progressed through the investigation of individual quantitative traits underlying disease risk- IOP, optic nerve cupping as measured by CDR, and CCT. For example, a recent study showed that both CDR and IOP have genetic components that correlate with POAG [44]. Wirtz et al. [15] proposed that searching for genes influencing POAG phenotype components may increase the power to dissect the genetic architecture of POAG. The question of whether the genetic etiology of POAG is determined by a large number of rare variants with major effects on the disease risk (rare variant, common disease hypothesis) or whether there are multiple common variants underlying the disease (common variant, common disease hypothesis) is also being addressed in new studies [45]. For example, based on a genome wide SNP analysis of a large cohort, Ramdas et al. [45] proposed a polygenic model for CDR.

Genes in which mutation causes anterior segment angle anomalies and glaucoma are strong candidates for glaucoma susceptibility and may contribute to glaucoma more frequently than expected, and possibly play an important role in the common form of POAG. In this study three candidate genes *TGFβ2*, *BMP4*, and *FOXC1* were examined. Selection of these candidates was based existing knowledge of their function in the anterior segment.

In this study, despite the evidence that the candidate genes are involved in glaucoma disease pathways no significant associations were identified between *TGFβ2*, *BMP4*, and *FOXC1* alleles and haplotypes and POAG in a population of patients and controls recruited form the North East of England. This represents the first association analysis of *TGFβ2*, *BMP4*, and *FOXC1*; the lack of association of common polymorphism does not provide evidence in support of the hypothesis that these genes play a significant genetic role in the pathogenesis of glaucoma among white British subjects.

A lack of association, however, should be interpreted with caution unless proven by investigating a substantially larger sample of the population. This is because of the small possibility of such results being caused by a false-negative error, which is confounded by the small size of the OHT sub-group. The key determinant of quality in an association study is the sample size since the power to detect an association depends partly on this as well as the size of the effect. If a study with negative results has insufficient power, an association is unlikely to be significant as there is a higher chance that it is a falsely negative result. For this study, with a sample size of 276 controls and 330 cases, an adequate study power of 80% was achieved if a difference in genotype and allele frequency was 10%–18% between controls and cases at a significance level of p<0.05. However, if the individual subgroups are considered, the OHT group (n=58) was clearly under-represented, despite being adequate to produce a robust result as being part of the whole cohort. In addition, the results obtained from this study reflect only one ethnic group (in this case white British adults) and not other ethnicities and it would need to be confirmed if other ethnic groups showed the same results.

One of the limitations of this study is the absence of CCT measurement. In the current study, IOP measurements were checked by a Tono-Pen which is less affected by CCT [46], in addition to performing applanation tonometry. Two recent studies that adjusted IOP for CCT found that the correction did not alter the diagnosis of HTG or NTG [47], and did not affect the relationship between the prevalence of POAG and IOP respectively [48]. Furthermore, to be certain that the participants were correctly assigned to the appropriate case groups, individuals with borderline IOPs (21–23 mmHg) were excluded from this study. Even if a correction formulae were to be applied with a 10 µm change in the corneal thickness inducing a 0.2 mmHg change in IOP reading [49], a 2–3 mmHg IOP change (which would include the excluded individuals within the borderline IOP) would induce a 100–150 µm change in the CCT, which is a considerable amount. Hence, it is still highly unlikely that the individuals with IOPs of 20 mmHg or below or IOPs of 24 mmHg or higher would have their diagnosis altered (assuming that the average CCT is approximately 537–550 µm) [50] since these subjects would be required to have either an abnormally thin corneas or an unusually thick corneas.

In summary, this study did not demonstrate any significant allelic or haplotype associations between *TGFβ2*, *BMP4*, and *FOXC1* and OHT/POAG. It is hence concluded that common variants in the *TGFβ2*, *BMP4*, and *FOXC1* genes do not play a major role in the genetic etiology of POAG in the population investigated.

## Appendix 1. Primer information.

To access the data, click or select the words “Appendix 1.” This will initiate the download of a compressed (pdf) archive that contains the file.
